# Systemic mechanisms of necrotic cell debris clearance

**DOI:** 10.1038/s41419-024-06947-5

**Published:** 2024-08-01

**Authors:** Sara Schuermans, Caine Kestens, Pedro Elias Marques

**Affiliations:** Laboratory of Molecular Immunology, Department of Microbiology, Immunology and Transplantation, Rega Institute for Medical Research, KU Leuven, Leuven, Belgium

**Keywords:** Cell death and immune response, Immune cell death

## Abstract

Necrosis is an overarching term that describes cell death modalities caused by (extreme) adverse conditions in which cells lose structural integrity. A guaranteed consequence of necrosis is the production of necrotic cell remnants, or debris. Necrotic cell debris is a strong trigger of inflammation, and although inflammatory responses are required for tissue healing, necrotic debris may lead to uncontrolled immune responses and collateral damage. Besides local phagocytosis by recruited leukocytes, there is accumulating evidence that extracellular mechanisms are also involved in necrotic debris clearance. In this review, we focused on systemic clearance mechanisms present in the bloodstream and vasculature that often cooperate to drive the clearance of cell debris. We reviewed the contribution and cooperation of extracellular DNases, the actin-scavenger system, the fibrinolytic system and reticuloendothelial cells in performing clearance of necrotic debris. Moreover, associations of the (mis)functioning of these clearance systems with a variety of diseases were provided, illustrating the importance of the mechanisms of clearance of dead cells in the organism.

## Facts

- Necrotic cell debris contains several DAMPs, which induce pro-inflammatory signaling through engagement of PRRs.
- Besides phagocytosis by leukocytes, systemic clearance mechanisms present in the bloodstream and vasculature are also involved in necrotic cell debris clearance.
- Extracellular DNases and the actin-scavenger system cooperate to facilitate efficient clearance of necrotic DNA and actin, with the latter ensuring that actin does not inhibit DNase activity.
- Necrotic cell debris can interact with fibrin and thereby increase resistance to fibrinolysis.
- Kupffer cells and LSECs are involved in the systemic clearance of necrotic cell debris due to their abundant expression of scavenging receptors for cell debris.

## Open questions

- Can the efficacy of DNase 1 treatment be augmented through integration of treatments with components of the actin-scavenger system?
- Does fibrin help or hinder recovery, considering its interactions with necrotic cell debris that may impede degradation through increased resistance to fibrinolysis?
- What are the respective roles of LSECs and Kupffer cells in the clearance of necrotic cell debris, their interplay and which receptors are involved in this process?

## Introduction to cell death

Cell death is a natural cellular process, inevitable for all living organisms. It is crucial for maintaining homeostasis and eliminating potentially harmful cells from our body. Cells have three main different ways to experience death. Under physiological conditions, cells die chiefly through a well-defined process of programmed cell death termed apoptosis, derived from the Greek word for “falling off” [1]. Morphologically, apoptotic cells exhibit cell shrinkage, chromatin condensation, nuclear fragmentation, membrane blebbing and the formation of apoptotic bodies, while the plasma membrane remains intact [2]. Biochemically, apoptosis is characterized by the activation of diverse apoptotic caspases (*e.g*. caspase 3) and caspase-dependent nucleases, resulting in widespread cleavage of cellular components [3]. Although apoptosis has been studied most extensively, cells also have other ways to die. Autophagy, meaning “eating of self”, is a process in which the cell degrades and clears damaged proteins and organelles, as well as intracellular pathogens (*e.g*. bacteria) from its cytoplasm in a lysosome-dependent manner [4]. Necrosis, the third main type of cell death, was initially identified as a type of uncontrolled cell death that lacks the features of apoptosis and autophagy [5]. It has long been considered only as a type of uncontrolled cell death induced by exposure to extreme stress or stimulation, such as thermal, mechanical and (bio)chemical insults. However, necrosis can also occur in a regulated manner, exemplified by necroptosis, ferroptosis, pyroptosis and NETosis. In general, necrosis is characterized by cellular swelling and irreversible loss of membrane integrity [6, 7].

Accidental cell death (unregulated necrosis), in contrast to apoptosis, is adenosine triphosphate (ATP)-independent and does not require energy [8]. When cells try to maintain ATP production, they switch to anaerobic glycolysis with the production of lactate, resulting in a pH decrease. The attempts to neutralize the pH decrease result in increased intracellular calcium levels. Eventually, the increased calcium levels lead to mitochondrial overload and altered bioenergetics, worsening the ion balance disturbance [9]. In addition to ATP production, mitochondrial respiration is also an important source of reactive oxygen species (ROS). ROS initiate damage to DNA, proteins and lipids, and as a result worsen the mitochondrial dysfunction [10]. Eventually, this culminates in osmotic lysis of the plasma membrane and exposure of intracellular molecules to the extracellular environment in a disorderly fashion, leading to the formation of necrotic cell debris.

Necroptosis, a type of regulated necrosis, is characterized by activation of receptor-interacting protein kinase 3 (RIPK3) by diverse stimuli. RIPK3 phosphorylates and activates mixed lineage kinase-like protein (MLKL), which in turn undergoes conformational change, oligomerization and translocation to the membrane. In the membrane, MLKL promotes both pore formation and the recruitment of ion channels, resulting in membrane permeabilization [11]. Pyroptosis is a form of pro-inflammatory regulated cell death often initiated upon inflammasome assembly. The activation of various caspases induces the cleavage of their respective gasdermins into active forms, resulting in the formation of plasma membrane pores and the rapid loss of membrane integrity. For example, gasdermin D is cleaved by caspase 1 and mouse caspase 11 or human caspase 4 and 5, whereas gasdermin E is processed by caspase 3 [12]. Ferroptosis is a mode of regulated cell death characterized by iron-mediated phospholipid peroxidation, in particular of polyunsaturated fatty acids. Glutathione depletion and increased ROS production result in diminished activity of glutathione peroxidase 4 (GPX4), which is a central regulator of ferroptosis. Amongst others, the accumulation of phospholipid hydroperoxides in cellular membranes causes rapid and irreparable membrane destruction, leading to cell death [13]. Finally, during NETosis, which was first described in neutrophils, web-like chromatin structures complexed with granular and cytoplasmic proteins known as neutrophil extracellular traps (NETs) are purposefully extruded into the extracellular environment to provide antimicrobial activity. Briefly, the generation of NADPH oxidase-dependent or mitochondrial ROS leads to activation of myeloperoxidase (MPO), neutrophil elastase (NE) and protein-arginine deiminase type 4 (PAD4), which in turn promote chromatin decondensation and disintegration of the granular membranes, culminating in NET release [14].

## Release of necrotic cell debris molecules

**Fig. 1:**
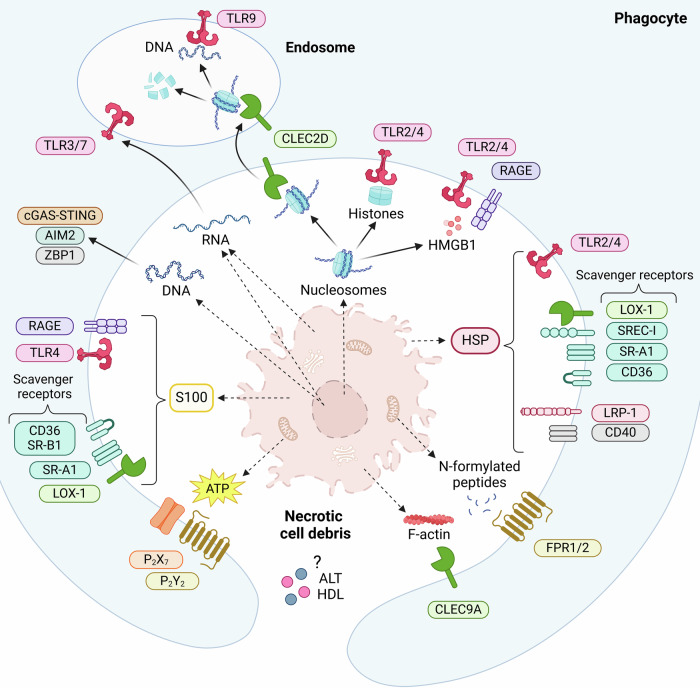
Necrotic cell debris and its phagocytic receptors. A plethora of damage-associated molecular patterns (DAMPs) are released by necrosis, including nuclear and mitochondrial DNA, RNA, histones, nucleosomes, HMGB1 (high mobility group box 1 protein), actin, *N*-formylated peptides, ATP (adenosine triphosphate), S100 proteins and HSPs (heat shock proteins). Their clearance is mediated by scavenger and pattern recognition receptors on phagocytes. TLR toll-like receptor, cGAS cyclic GMP-AMP synthase, STING stimulator of interferon genes, AIM2 absent in melanoma 2, ZBP1 Z-DNA-binding protein 1, FPR formyl peptide receptor, CLEC C-type lectin receptor, RAGE receptor for advanced glycation end products, SR scavenger receptor, CD cluster of differentiation, LOX-1 lectin-like oxidized low-density lipoprotein receptor-1, SREC-I scavenger receptor expressed by endothelial cells I, P_2_X/Y purinergic P_2_X or P_2_Y receptors, LRP-1 low-density lipoprotein receptor-related protein-1.

Necrotic cell debris is composed largely of damage-associated molecular patterns (DAMPs) and is able to induce inflammation through engagement of pattern recognition receptors (PRRs) such as toll-like receptors (TLRs) and C-type lectin receptors [15]. These DAMPs are normally concealed inside the cell, and comprise a variety of molecules, among which DNA, RNA, histones, actin, *N*-formylated peptides, high mobility group box 1 protein (HMGB1) and ATP are some well-established ones [16]. Moreover, the enzymes lactate dehydrogenase (LDH) and alanine transaminase (ALT) that can be found in the cytoplasm of all cells and hepatocytes, respectively, are examples of DAMPs that are often used as clinical parameters for organ injury [17, 18]. Although inflammatory responses are required for tissue healing, the debris may lead to excessive inflammation and significant collateral damage. Figure 1 provides a summary of the DAMPs found within necrotic cell debris, along with their corresponding receptors. Box 1 delineates the specific DAMPs released during different regulated necrotic cell death modalities. However, it is important to note that our understanding of these DAMPs is still limited, as not all aspects are fully known. The next paragraphs discuss the release of necrotic cell debris molecules in general during injury.

Box 1 Release of DAMPs in different regulated necrotic cell death modalities **Necroptosis:** Several DAMPs released by necroptotic cells have been documented in the literature. Among these, HMGB1 is the best characterized and known to promote inflammation both in vitro and in vivo. Other DAMPs released by necroptotic cells include the S100 protein S100A9 and ATP, although the specific roles of these molecules in necroptosis are not well understood [182–184].

**Pyroptosis:** Similarly to necroptosis, HMGB1 released by pyroptotic cells promotes inflammation by activating NF-κB through TLR4 and RAGE [183–185]. Since HMGB1 can bind DNA and other negatively charged molecules, it can synergize with their pro-inflammatory effects [186]. However, there is currently no literature documenting the release of DAMPs other than HMGB1 during pyroptosis.

**Ferroptosis:** The release of DAMPs in ferroptosis remains an understudied topic. Again, HMGB1 has been identified as an important DAMP released following ferroptotic signaling [187]. While it is suggested that cell-free DNA is released during ferroptosis, clear evidence supporting this is currently lacking [184].

**NETosis:** NETs consist of chromatin complexed with granular and cytoplasmic proteins. Therefore, during the formation of these extracellular traps, DNA, histones, HMGB1 are released as DAMPs [188, 189], along with enzymes from the primary (e.g. elastase and myeloperoxidase), secondary (e.g*.* lactoferrin) and tertiary granules (e.g. gelatinase and matrix metalloproteinases) [188].

### Chromatin and histones

DNA is a negatively charged double-stranded polymer, which exists in the nuclear and mitochondrial compartments in mammals. Nuclear DNA is compacted with histones in the form of chromatin, whereas mitochondrial DNA is found inside the organelle as a circular chromosome. To compact DNA as chromatin, the positively charged histones form histone octamers (two copies of H2A, H2B, H3, and H4) that are wrapped 1.75 times by 147 base pairs of double-stranded DNA, resulting in nucleosome formation. Linker DNA interconnects the different nucleosomes, and binding of the linker histone H1 at 1:1 stoichiometry to nucleosomes results in both stabilization and organization in higher-order chromatin filaments [19]. Following cell death, the different chromatin components are released into the extracellular environment. The presence of circulating nuclear DNA, histones and nucleosomes following injury has already been extensively documented in the literature. Critically ill trauma patients that developed acute kidney injury presented increased circulating levels of both DNA and nucleosomes [20]. Examples of other inflammatory conditions during which these DAMPs are released include traumatic brain injury [18, 21], burn injury [22], acute lung injury [23], ischemia-reperfusion injury [24] and the chronic condition systemic lupus erythematosus (SLE) [25]. Interestingly, the immunostimulatory capacity of these DAMPs depends on the form in which they circulate [26]. The major nucleic acid sensors involved in DNA recognition are TLR9, cyclic GMP-AMP synthase (cGAS)-stimulator of interferon genes (STING), absent in melanoma 2 (AIM2) and Z-DNA-binding protein 1 (ZBP1), with the three latter ones being cytoplasmic DNA sensing mechanisms [27, 28]. Activation of these receptors results in the production of pro-inflammatory cytokines and type-I interferons [27]. Remarkably, the immune response against extracellular DNA through TLR9 has been reported to depend on the presence of histones. More specifically, the receptor CLEC2D recognizes and targets nucleosomes through the poly-basic sequences in the histone tails for DNA sensing by endosomal TLR9 [29]. Extracellular histones induce cytotoxicity and pro-inflammatory signaling via TLR2 and TLR4, a process that was enhanced by binding to DNA [30, 31]. Besides their inflammatory effect, histones also have cytotoxic effects due to the abundance of the positively charged lysine and arginine residues. These basic amino acid residues allow histones to interact with negatively charged cellular membranes and cause lysis. In contrast, histones in the form of nucleosomes do not possess cytotoxic properties [26, 32].

### Mitochondrial DNA

Given the mitochondrion’s evolutionary origins as bacterial symbiont, its components retain strong immunostimulatory properties and play an important role as potent DAMPs. In this regard, mitochondrial DNA (mtDNA) is well characterized. In the mammalian genome, the methylation of CpG islands is an epigenetic phenomenon, and due to its bacterial origin, mtDNA exhibits a distinct methylation pattern compared to nuclear DNA, causing it to activate multiple PRRs of the innate immune system and induce inflammation. Unmethylated or hypomethylated CpG repeats of mtDNA are detected by TLR9, whereas double-stranded mtDNA is sensed by the cGAS-STING pathway [33]. Cellular stress triggers mitochondrial damage and enhances production of mitochondrial ROS, resulting in the release of oxidized mtDNA. In a mouse model of lupus, the extracellular release of oxidized mtDNA has been shown to be pathogenic through STING signaling, which was inhibited by administration of mitochondrial ROS scavengers [34]. In addition, oxidized mtDNA has been shown to lead to inflammasome activation via NLR family pyrin domain-containing protein 3 (NLRP3), NLR family CARD domain-containing protein 4 (NLRC4) or AIM2, although the exact mechanisms remain elusive [33]. Other conditions characterized by an increase in circulating mtDNA include trauma [35], acute liver injury [36], and acute brain injury [21].

### *N*-formylated peptides

Besides mtDNA, mitochondrial *N*-formylated peptides (mtFPs) are another type of mitochondrial DAMP. MtFPs are recognized by formyl peptide receptors (FPRs), which are G protein-coupled receptors that exhibit different binding affinities for mtFPs depending on their origin, composition and length. For example, mtFPs containing a methionine residue at the 5′ terminus [*e.g. N*-formyl-Met-Leu-Phe (fMLF)] have a high affinity for FPR1, whereas FPR2 is more selective for mtFPs with a positive charge at the C-terminus [*e.g. N*-formyl-Met-Leu-Phe-Lys (fMFLK)] [37]. Binding of mtFPs to FPR1 on the plasma membrane of neutrophils contributes to their migration into injured tissues [36, 38]. Moreover, activation of FPR1 resulted in desensitization and partial internalization of CCR1, but not CCR2, suggesting FPR1 has the ability to control migration by certain chemoattractants [39]. Examples of conditions during which levels of mtFPs were increased include trauma [40] and sepsis. In the case of sepsis, high values of mtFPs were strongly associated with secondary infections and increased mortality [41]. In addition to mtFPs, FPR2 is most known for binding pro-resolving lipid mediators, including lipoxins (LXA_4_) and resolvins (RvD1 and RvD3), assisting in dampening inflammation by limiting leukocyte migration and inducing efferocytosis [42].

### Actin

Actin is the most abundant protein in mammalian cells and a major cytoskeletal component, performing many essential functions for mechanical support and cell movement [43]. Actin undergoes cycles of polymerization and disassembly between its globular (G-actin) and filamentous (F-actin) form. During necrotic cell death, cells expose both G- and F-actin following plasma membrane disruption, after which F-actin but not G-actin is recognized by the C-type lectin receptor CLEC9A (DNGR-1) on dendritic cells [44, 45]. Increased levels of circulating actin have been associated with diverse conditions in patients, including severe trauma [46], sepsis [47], and burn injury [22]. Moreover, actin was detected in the synovial fluid of rheumatoid arthritis patients [48] and as part of the highly viscous lung secretions in cystic fibrosis (CF) patients. More specifically, DNA and actin are released from necrotic cells (or NETs) and form bundles, altering the viscoelastic properties of the sputum. This results in difficulty to clear infected airway fluid, worsening CF pathology [49]. Additionally, it was shown that histones are able to bundle actin filaments by electrostatic and hydrophobic interactions, affecting the actin structure in the sputum of CF patients, which also contributes to the disease [50].

### HMGB1

HMGB1 is a non-histone nuclear protein, which binds linker DNA through its two DNA-binding regions and histones through its acidic tail [51]. In the nucleus, similar to histones, HMGB1 aids in modulating the chromatin structure and regulating gene transcription. HMGB1 release was reported during various types of cell death and to be increased in patients with ischemia-reperfusion injury [24, 52] and burn injury [53]. Moreover, the release of HMGB1 after trauma has been associated with injury severity and the activation of the complement system [54]. Interestingly, the oxidation state of HMGB1 results in diverse inflammatory activities. For instance, reduced HMGB1 forms a heterocomplex with CXCL12 to enhance migration via CXCR4, but fully oxidized HMGB1 enhances immune tolerance. In addition, through binding to TLR2 or TLR4, or internalization via receptor for advanced glycation end products (RAGE), HMGB1 induces cytokine production and inflammation [55].

## Mechanisms of necrotic debris clearance

**Fig. 2:**
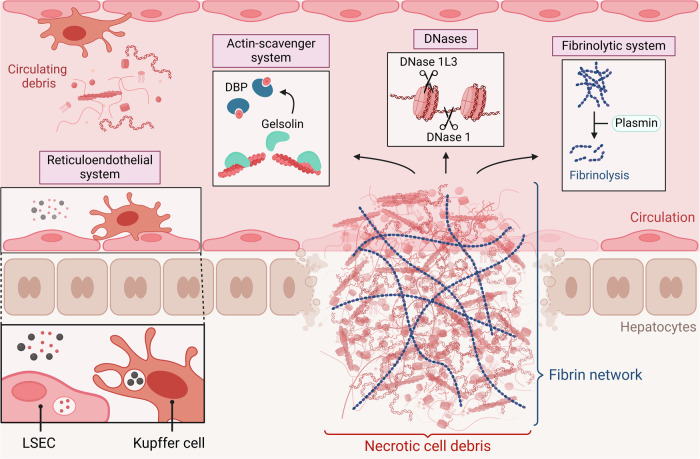
Overview of the different systemic clearance mechanisms of necrotic cell debris. Different mechanisms cooperate to facilitate the clearance of necrotic cell debris, as illustrated here by liver injury. These mechanisms include: 1) The removal of necrotic DNA mediated by the circulating deoxyribonucleases DNase 1 and DNase 1L3; 2) Scavenging of necrotic actin by the actin-scavenger system, specifically by vitamin D-binding protein (DBP) and gelsolin; 3) Fibrinolysis by plasmin of the fibrin network bound to necrotic cells; 4) Clearance of circulating necrotic cell debris by the reticuloendothelial system, comprising both the Kupffer cells and the liver sinusoidal endothelial cells (LSECs).

Necrosis can be distinguished from apoptosis both biochemically and morphologically. The conversion of apoptotic cells into apoptotic bodies requires energy and enzymatic activity from caspases. On the other hand, necrosis is characterized by catastrophic loss of energy and cellular integrity [16]. These differences suggest that the clearance of the necrotic cell debris may differ from the clearance of apoptotic bodies. The apoptotic bodies, which are composed of closely packed organelles, cytoplasm and often nuclear fragments [56], are cleared by resident phagocytes (*e.g*. macrophages) and parenchymal cells by a process termed efferocytosis (“carrying to the grave”) [16, 57]. Although general phagocytosis and efferocytosis are mechanistically similar processes, their outcome is different. Phagocytosis of bacteria or necrotic cells triggers a pro-inflammatory response, whereas efferocytosis triggers resolution of inflammation, regeneration of functional tissue and restoration of homeostasis [58]. Whereas apoptotic bodies are completely engulfed during efferocytosis, necrotic cell debris was shown to be cleared by a process resembling macropinocytosis in vitro, which involves the co-ingestion of extracellular fluid and necrotic target material [59, 60]. Besides local phagocytosis by leukocytes, there is accumulating evidence that systemic mechanisms are also involved in necrotic cell debris clearance. In this review, we focus on systemic clearance mechanisms present in the bloodstream and vasculature that often cooperate to drive the removal of necrotic cell debris, as illustrated in Fig. 2.

### Deoxyribonucleases (DNases)

In general, the removal of extracellular DNA is essential to limit inflammation and to prevent autoimmunity, which is primarily achieved through DNA cleavage by DNases or capture by macrophages. Hence, DNases play a crucial role in the intra- and extracellular clearance of DNA during physiological processes, such as cell differentiation, but also following excessive cell death [61]. The DNase 1 protein family comprises DNase 1 along with its three related nucleases DNase 1-like 1 (1L1), DNase 1L2, and DNase 1L3, all characterized by similar biochemical properties. More specifically, their nuclease activity requires the presence of Ca^2+^ in combination with Mg^2+^, Co^2+^ or Mn^2+^ as co-factor. The specific combination of the co-factor and DNase dictates its pH optimum, ranging from pH 5 to pH 9, but in general, a neutral pH of 7–7.5 [62]. DNase 1 protein family members are endonucleases and cleave double-stranded DNA into two oligonucleotides with 5’-phospho and 3’-hydroxy ends, resulting in the formation of di-, tri- or tetra-oligonucleotides [63].

DNase 1 and DNase 1L3 are the extracellular DNases responsible for the majority of DNase activity in the circulation [64–66]. DNase 1 is produced by a variety of endocrine and exocrine glands that line the gastrointestinal and urogenital tract (*e.g*. the liver, parotid gland, and kidney). This broad expression profile causes DNase 1 to be the major nuclease found in almost all mammalian body fluids, such as blood, urine, and saliva [67, 68]. DNase 1L3 is also known as DNase γ, DNase Y, nh-DNase or LS-DNase, and is secreted by mononuclear phagocytes, more specifically dendritic cells and macrophages in the liver, spleen and intestine [69]. In terms of substrate specificity, DNase 1 preferentially cleaves double-stranded “naked” linker DNA, whereas DNase 1L3 is more effective against membrane- and/or protein-associated DNA (*e.g*. chromatin) [70–72].

**Fig. 3:**
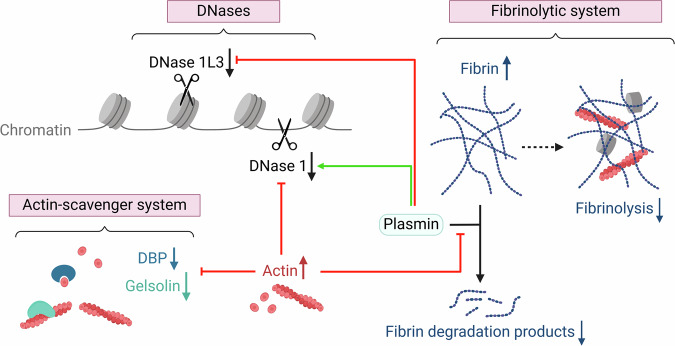
The interplay between DNases, the actin-scavenger system and the fibrinolytic system during injury. The interplay between the diverse mechanisms facilitating the clearance of necrotic cell debris is complex. During injury, the actin-scavenger system can become saturated, increasing circulating actin levels. This results in the inhibition of DNase 1 activity, thus impeding the internucleosomal cleavage of necrotic DNA. The activity of the fibrinolytic system, particularly plasmin, can be altered depending on the severity of injury. Plasmin enhances DNase 1 activity while hampering DNase 1L3 activity through the degradation of DNA-binding proteins. Histones and actin can both interact with fibrin, increasing resistance of the fibrin network to fibrinolysis. Additionally, actin acts as a noncompetitive inhibitor of plasmin, further reducing fibrinolysis.

Although the core domains of both DNases resemble each other, there are some key structural differences that explain their enzymatic activity. First, actin has the well-known ability to inhibit DNase 1 [73] (Fig. 3). DNase 1 and monomeric G-actin bind with high affinity by forming a 1:1 stochiometric complex, thereby inhibiting both its nuclease activity and G-actin polymerization [74, 75]. Moreover, DNase 1 binds to F-actin and induces its depolymerization to G-actin [75]. In contrast, DNase 1L3 is naturally resistant to actin inhibition due to multiple differences in the actin recognition site. Determinants for actin resistance include electrostatic repulsion between DNase 1L3 and actin, as well as the lack of stabilizing salt bridges between both molecules [76]. Second, previous research suggested that the specific activity of DNase 1L3 could be attributed to its C-terminal domain (CTD) [69]. McCord et al. identified the CTD to be a positively charged polypeptide that extends from the enzymatic core and possesses conformational versatility. Interestingly, they found that the CTD interacts with DNA to increase the net affinity of DNase 1L3 for its DNA substrate, and thereby allows DNase 1L3 to interact with a diversity of DNA complexes [76]. However, Englert et al. discovered by engineering a dual-active DNase with combined DNase 1 and DNase 1L3 activities that the ability of DNase 1L3 to degrade chromatin is embedded into three distinct areas of the enzyme’s core body. Two of these domains correspond to actin recognition sites in DNase 1, which are absent in DNase 1L3. The third domain in DNase 1L3 contains three additional lysine residues compared to DNase 1, resulting in an increase in both its DNA-binding affinity and DNA-degrading activity [72].

During apoptosis, DNA is cleaved by the nucleases apoptosis-inducing factor (AIF), endonuclease G and caspase-activated DNase (CAD) [77]. However, necrotic cell death does not typically involve the activity of these intracellular nucleases. In addition, under physiological circumstances, three prime exonuclease 1 (TREX1) is involved in the degradation of cytosolic DNA, but oxidized DNA is resistant to TREX1-mediated degradation, resulting in lack of DNA fragmentation in cells that undergo necrosis [78]. Therefore, the burden of clearing necrotic DNA is likely left to DNase 1 and DNase 1L3 after necrotic cells lose plasma membrane integrity. Although these circulating DNases are abundantly present, necrotic DNA accumulates in tissue injury sites for days [79, 80]. Delayed DNA degradation plays an important role during injury, and may be caused by either genetic defects in the production of DNases, or inhibition of DNase activity by molecules that are released into the bloodstream. Table 1 summarizes a list of acute injury conditions in which DNase protein levels or activity were altered. Overall, DNase activity is reduced in blood upon a variety of injuries, both in patients and mice.

**Table 1:** Sterile acute inflammatory conditions and their impact on DNases.

Disease/condition	Species	Effect		References
		Blood levels	Activity	
Acute kidney injury	Human	↑ DNA	= DNase activity	[144]
Acute liver failure	Mouse	↑ DNA	↓ DNase activity	[145]
Acute thrombotic microangiopathy	Human	↑ DNA	↓ DNase activity	[146]
Burn	Human	↑ DNA	↓ DNase activity	[22]
		↑ actin		
		= DNase protein levels		
	Mouse	↑ DNA	-	[147]
		↑ DNase protein levels		
Stroke	Human	↑ DNA	↓ DNase activity	[148]
Trauma	Human	↑ DNA	↓ DNase activity	[18, 35, 149]
		↑ actin		
		↑ DNase protein levels		

-: Not determined.

As mentioned above, an initial reason for problematic DNA degradation is due to deficiencies in DNase production. For example, mice that lack DNase 1 exhibited delayed chromatin clearance after the injection of purified chromatin [81]. Moreover, DNase 1 and DNase 1L3 have been reported to degrade NETs following neutrophilia and sepsis, and deficiencies in the production of both DNases resulted in intravascular clot formation by these traps, which led to blood vessel obstruction and severe organ damage [64]. Importantly, removal of DNA released during cell death is essential to avoid autoimmunity. SLE is a chronic autoimmune disease characterized by the production of anti-nuclear antibodies (*e.g*. anti-DNA autoantibodies) [82]. DNase 1- and DNase 1L3-deficient mice develop SLE-like disease, characterized by the presence of anti-nuclear antibodies [69, 83], and mutations in DNase 1 and DNase 1L3 have been associated with this condition in humans [84, 85]. Notably, the reduction in DNase activity could also be attributed to the generation of anti-DNase 1 antibodies. These antibodies were identified in both SLE and Sjögren’s syndrome, another autoimmune disease that is also characterized by diminished DNase 1 activity [82].

A second reason for reduced DNase activity is the occurrence of nuclease inhibition in serum. DNase 1 is inhibited by actin, which is found elevated in the bloodstream in a number of diseases including trauma and burn injury. Similarly, DNase 1L3 activity is inhibited due to proteolysis by plasmin, also commonly activated during injuries [66]. Interestingly, DNase 1 and the plasminogen system work together in vitro to facilitate chromatin breakdown by simultaneously degrading DNA and DNA-binding proteins. More specifically, proteolysis by plasmin facilitates internucleosomal chromatin breakdown by the cleavage of linker histone H1 [65]. In addition, the displacement of DNA-binding proteins from chromatin was mimicked by the addition of heparin [66, 81]. In contrast to the plasminogen system, heparin displaces both core and linker histones, and thus results in random chromatin cleavage. Although heparin and the plasminogen system improved chromatin degradation by DNase 1, both inhibited chromatin degradation by DNase 1L3 [66] (Fig. 3).

Numerous studies have reported DNase use as a promising treatment for pulmonary diseases, in particular for CF. However, the presence of actin in, for example, the sputum of CF patients can inhibit DNase 1-mediated DNA degradation, impeding its therapeutic effect in CF and other diseases. To minimize actin inhibition, DNase 1 was reengineered as Pulmozyme (dornase alfa). However, the addition of either gelsolin, which enhances actin depolymerization, or polyanions to disrupt the electrostatic interactions within the polymers further improved its DNase efficacy, indicating that inhibition by DNA-actin complexes still remains [49]. Dornase alfa has been approved for the treatment of CF, where it led to improved lung function and decreased pulmonary exacerbations (*i.e*. periods with breathing difficulties) [86]. Interestingly, the subtype DNase 1L2 is resistant to actin inhibition and showed 4-fold higher potency in reducing viscosity of actin-containing artificial mucus compared to dornase alfa [87]. Besides positive effects in CF, it has been proven that DNase 1 treatment reduces inflammation in multiple animal models of disease [88]. Table 2 summarizes a list of murine models in which DNase 1 was used as a treatment. In general, DNase 1 treatment reduced levels of circulating DNA, decreased pathology and inflammation.

**Table 2:** Effects of DNase 1 treatment in murine models.

Mouse model	Key findings	References
Acetaminophen-induced acute liver injury	↓ hepatotoxicity	[79]
	↓ intravascular DNA deposition	
	↓ circulating TNF-α and CXCL1 levels	
	↓ liver neutrophil recruitment	
Acute respiratory distress syndrome	↓ neutrophil infiltration in lungs	[150]
	↓ NETs in lungs, BALF and blood	
	↓ platelet-NET aggregates in lungs	
	↓ platelet activation in lungs, BALF and blood	
	↓ IL-6 and IL-17 expression in lungs, BALF and blood	
	↓ pulmonary tissue hypoxia	
	↑ lung perfusion	
B16 melanoma	↓ circulating DNA levels	[151]
	↓ surface lung metastases	
	↓ internal lung metastases	
	↓ liver metastases	
	↑ disintegration of NETs in metastatic foci	
Cecal ligation and puncture-induced sepsis	↓ circulating DNA levels	[152]
	Early administration:	
	↑ circulating IL-6 and IL-10 levels	
	↑ infiltrating inflammatory cells in lungs and kidneys	
	↑ organ damage in lungs and kidneys	
	Late administration:	
	↓ circulating IL-6 levels	
	↓ neutrophil infiltration in lungs	
	↓ organ damage in lungs, kidneys and liver	
	↑ circulating IL-10 levels	
	↑ survival	
Chronic stress	↓ neuroinflammation	[153]
	↓ social behavior deficits	
Intestinal ischemia-reperfusion-induced acute lung injury	↓ pulmonary edema/protein exudation	[154]
	↓ atelectasis	
	↓ interstitial inflammation	
	↓ histopathological score	
	↓ protein influx into the alveoli	
	↓ DNA levels in blood and BALF	
	↓ NETs in lungs and BALF	
	↓ IL-6 and TNF-α expression in lungs	
	↓ CXCL1 and CXCL2 levels in BALF	
	↑ survival	
Ischemic stroke	↓ infarct volume	[155]
	↑ functional outcome (neurological and motoric behavior)	
Limb ischemia-reperfusion injury	↓ ETs in skeletal muscle	[156, 157]
	↓ muscle fibrosis	
	↓ infiltrating inflammatory cells (Ly6B.2^+^)	
	↑ limb perfusion	
	↑ angiogenesis in ischemia-reperfusion area	
	↑ recovery of motor function	
Myocardial infarct	↓ circulating DNA and nucleosome levels	[158]
	↑ left ventricular remodeling	
	↑ cardiomyocyte survival	
Subarachnoid hemorrhage	↓ NETs in cerebral cortex	[159]
	↓ microthrombosis	
	↓ neurological deficits	
	↓ brain edema	
	↓ neuronal injury	
	↑ cerebrospinal fluid flow	
	↑ restoration of early cortical perfusion	
	↑ blood-brain barrier integrity	
Traumatic brain injury	↓ lesion volume	[160]
	↓ brain edema	
	↓ clotted fibrin deposition	
	↓ activated microglia	
	↑ blood-brain barrier integrity	
	↑ cerebral perfusion	

### The actin-scavenger system

The sequestration of actin released into the extracellular environment is essential to avoid organ damage and to restore DNase activity. However, the relationship between chromatin, actin, DNases and the actin-scavenger system is complex due to their dynamic interactions. The extracellular actin-scavenger system consists of two plasma proteins, vitamin D-binding protein (DBP) or Gc-globulin, and gelsolin, whose combined action protects the organism from the deleterious effects of extracellular actin polymerization [89]. Gelsolin was first described as a cytoplasmic actin-binding protein, composed of six homologous domains and three actin-binding sites: two monomer-binding sites and one filament-binding site. Further studies revealed a secreted isoform of gelsolin that was identified to be structurally similar but not identical to cytoplasmic gelsolin [90]. The majority of plasma gelsolin is produced by skeletal muscle [91] and it is involved in severing F-actin to G-actin extracellularly, thus, disassembling F-actin fragments in plasma. The resulting G-actin monomers are bound by DBP, which releases gelsolin from actin. DBP, a 58 kDa multifunctional plasma protein mainly synthesized by the liver [92], forms high affinity (*K*_d_ = 10^−9^ M) 1:1 stochiometric complexes with monomeric G-actin, also preventing actin polymerization [93]. Subsequently, the DBP-actin complex is rapidly cleared from the circulation (half-life of ≈ 30 min) by the hepatic reticuloendothelial system, which consists of the liver’s sinusoidal endothelial cells, as well as the tissue-resident Kupffer cells [94]. Thus, the two proteins work in a concerted fashion in circulation, with gelsolin depolymerizing actin filaments and DBP complexing and targeting actin for clearance (Fig. 3).

It has been shown that injury often results in overloading of the actin-scavenger system, which becomes quickly saturated. More specifically, injection of monomeric G-actin into the bloodstream of rats resulted in a decrease in the plasma levels of the actin-scavenger system molecules and polymerization of G-actin into F-actin in the vasculature. This led to intravascular obstruction by the filaments and endothelial injury in the pulmonary circulation [95]. A summary of conditions in which the actin-scavenger system was affected has been listed in Table 3. Acute tissue injury settings, such as acute liver failure [96, 97], burn injury [22] and traumatic brain injury [18], are accompanied by a reduction in plasma gelsolin and/or DBP levels. Even during chronic conditions, such as rheumatoid arthritis and multiple sclerosis, plasma gelsolin levels were reduced [48, 98]. Moreover, the actin-scavenger system molecules may also be released into other body fluids, where actin is exposed, and form complexes to neutralize its toxic effects. The presence of actin and gelsolin-actin complexes in synovial fluids of rheumatoid arthritis patients corroborates the local consumption of the potentially anti-inflammatory gelsolin in the inflamed joint [48]. Furthermore, following burn injury, actin complexed with gelsolin or DBP was detected in burn wound fluid [99].

**Table 3:** Inflammatory conditions and the impact on the actin-scavenger system.

Disease/condition	Species	Levels in		References
		Blood	Other body fluids	
Acute liver failure	Human	↓ DBP	-	[96, 97]
Acute lung injury	Mouse	↓ gelsolin	-	[161]
Bronchopulmonary dysplasia	Mouse	-	BALF	[103]
			↑ F-actin	
			↑ F-actin/gelsolin ratio	
	Human	-	↑ F-actin/gelsolin ratio	
Burn	Human	↓ gelsolin	Burn wound fluid	[22, 99]
		↓ DBP	Detection of actin, gelsolin and DBP	
		↑ actin		
Multiple sclerosis	Human	↓ gelsolin	Cerebrospinal fluid	[98**]**
		= DBP	= gelsolin	
			= DBP	
Rheumatoid arthritis	Human	↓ gelsolin	Synovial fluid	[48]
		↑ actin	Detection of actin and gelsolin	
Sepsis	Mouse	↓ gelsolin	-	[102**]**
		↑ actin		
	Human	↓ gelsolin	-	[47]
		↑ actin		
Traumatic brain injury	Human	↓ gelsolin	-	[18]
		↓ DBP		
		↑ G-actin		
Trauma	Human	↓ gelsolin	-	[100, 162, 163]
		↓ DBP		

-: Not determined.

Widespread release of actin and depletion of the extracellular actin-scavenger system proteins can be correlated with poor outcomes in diverse clinical conditions, such as trauma [100]. In intensive care unit patients, lower serum gelsolin levels were correlated with a deceased chance of discontinuation from mechanical ventilation within 28 days. Furthermore, these patients were less likely to be discharged from intensive care by day 14 compared to patients with higher baseline gelsolin levels [101]. In addition, children with acute liver failure presented reduced serum DBP levels, and patients with poorer outcomes generally had lower DBP levels than those with more favorable prognoses. In this way, serum DBP level is a promising marker to identify high-risk patients [97]. Taken together, these findings support the potential use of actin-scavenging molecules as therapeutic agents for a plethora of clinical conditions.

The treatment with components of the actin-scavenger system is less studied than treatment with DNases. In two murine models of sepsis (endotoxemia and cecal ligation/puncture), repletion of plasma gelsolin led to solubilization of circulating actin aggregates and a reduction in mortality. Additionally, plasma gelsolin treatment promoted an anti-inflammatory shift in endotoxemic mice, characterized by elevated IL-10 levels [102]. In the context of neonatal mice exposed to hyperoxia, serving as a murine model of bronchopulmonary dysplasia, F-actin promoted pro-inflammatory signaling through CLEC9A on CD103^+^ dendritic cells. Treatment with gelsolin, and thus F-actin scavenging, effectively blocked neonatal hyperoxia-induced inflammatory responses to rhinovirus infection, in particular by inhibiting IL-12, TNF-α and IFN-γ production. Moreover, gelsolin blocked hyperoxia-induced CD103^+^ dendritic cell expansion, further preventing inflammatory responses, and aided in preserving alveolarization in the mice [103]. As described before, DNase is known as a highly effective and well-tolerated mucolytic agent in CF. When gelsolin was added to DNase 1 treatment, a synergistic effect was observed, resulting in a two-fold higher rate of sputum fluidization [104]. This is supported by the observation that gelsolin activated DNase 1 by disassembling the actin-DNase 1 complex [105].

### The fibrinolytic system

The acute-phase response involves the synergistic activation of inflammation and coagulation, and is a common pathological feature of injury. During injury, vascular rupture and increased permeability allow hemostatic factors to enter the damaged tissue and initiate tissue repair [106]. The intrinsic and extrinsic coagulation pathways culminate in the activation of prothrombin into thrombin, which converts fibrinogen into fibrin that serves as a scaffold for thrombus formation [107]. Thus, fibrin deposition within damaged tissues is a common feature in numerous diseases, such as inflammatory arthritis, colitis, neuroinflammatory and musculoskeletal disease, bacterial infection and chemical liver injury [108]. Alongside coagulation, the fibrinolytic system is initiated to drive plasmin activation and destroy the fibrin clot, balancing out the vascular occlusion and promoting injury resolution. In more detail, the zymogen plasminogen is activated into plasmin, a serine protease responsible for fibrin degradation [109]. The conversion of plasminogen to plasmin is primarily mediated by tissue or urokinase plasminogen activators (tPa or uPA), while inhibition of fibrinolysis is predominantly governed by three inhibitors: plasminogen activator inhibitor type-1 (PAI-1), α2-antiplasmin and thrombin activatable fibrinolysis inhibitor (TAFI). Active PAI-1 regulates plasminogen activation by direct binding to free tPa, resulting in the formation of tPa-PAI-1 complexes and the subsequent loss of activity of tPa and plasmin. α2-antiplasmin prevents fibrin breakdown by binding irreversibly to the active site of plasmin [110]. Thrombin or thrombin-thrombomodulin complexes inhibit fibrinolysis by activating TAFI, which cleaves C-terminal lysine residues of fibrin and results in decreased accumulation of plasminogen and thus fibrinolysis [111].

During mild injury, plasmin is activated at the site of damage, where it is involved in fibrinolysis and resolving inflammation. However, during severe injury, plasmin activation increases damage by excessive fibrinolytic activity [46]. In addition, plasmin has a wide range of inflammatory actions, including matrix metalloproteinase (MMP) and complement activation, potentiating TLR signaling and NF-κB-mediated cytokine activation [107]. Furthermore, in burn injury patients, plasmin activation is a key component of the systemic cytokine storm, and inhibition of plasmin limits the tissue-damaging inflammation following injury. This was illustrated in a mouse model of burn injury, by a reduction in both plasma IL-6 levels and the neutrophil-to-lymphocyte ratio [112]. Nonetheless, plasmin could also play a crucial role in facilitating tissue healing through the elimination of necrotic cell debris. For example, in a mouse model of drug-induced liver injury, plasmin inhibition completely prevented upregulation of pro-inflammatory cytokines (*i.e*. TNF, CXCL1 and CXCL2) and reduced serum CCL2 protein levels. This was confirmed by treatment of Kupffer cells and bone marrow-derived macrophages with plasmin, which resulted in increased expression of these mediators and formation of pseudopodia. Furthermore, co-treatment with HMGB1 and plasmin showed a synergistic effect on plasmin’s cytokine upregulation. Crucially, inhibition of plasmin prevented clearance of necrotic cells from the liver as late as 72 hours, as well as monocyte and macrophage trafficking into the injured liver, indicating that plasmin was implicated in immune cell recruitment and recovery in injured sites [113]. Finally, in mouse models of traumatic and ischemia-reperfusion injury in the liver, plasminogen played a central role in macrophage and neutrophil recruitment to the damaged tissue [114]. In essence, evidence suggests that plasmin activation fuels the innate immune response during injury and indirectly facilitates the clearance of necrotic cell debris. However, plasmin was also shown to have significant direct effects on necrotic cells. As previously mentioned, plasmin collaborates with DNase 1 to degrade chromatin. More specifically, plasmin degrades the linker histone H1, thereby enhancing DNase 1 access to “naked” DNA for more efficient cleavage [65]. Moreover, it was shown by Samson et al. that cell death and loss of plasma membrane integrity lead to protein misfolding and crosslinking via disulfide bonding. The crosslinking and aggregation of cellular proteins including tubulins, HSPs, actin, and GAPDH led to the binding and activation of tPA, which in turn activated plasmin on necrotic cells. Proteolytic cleavage by plasmin was demonstrated to be central to degrade cellular debris in vitro in multiple cell types. Importantly, this mechanism was validated in vivo in plasminogen-deficient mice, which presented elevated levels of insoluble debris in the brain after traumatic brain injury [115, 116]. In addition, activation of plasmin on necrotic cells increased phagocytosis by monocyte-derived dendritic cells. This effect required plasmin to be proteolytically active but not to degrade the target, since phagocytosis of plastic microparticles was also increased in the presence of active plasmin. Interestingly, plasmin-treated dendritic cells retained an immature phenotype and produced more TGF-β, indicating that plasmin activity on necrotic debris assists its removal while restraining the capacity of the debris to induce an immune response by dendritic cells [117].

Hyperfibrinolysis (*i.e*. excessive fibrinolytic activity) is characteristic of early phases of severe injury, nonetheless, it was shown that fibrinolysis is also inhibited later “beyond a physiologic level” [118]. Severe blunt trauma in rats was found to induce a significant increase in plasma tPa levels, overwhelming its inhibition by PAI-1. Afterwards, plasma PAI-1 levels increased exponentially, restoring the balance between tPA and PAI-1 in the plasma [119]. This was confirmed in patients suffering from traumatic brain injury, in which hypercoagulability, fibrinolysis and fibrinolysis shutdown were activated consecutively [118]. Because actin is released from dying cells, it may be trapped in fibrin clots that form at sites of tissue injury. More specifically, F-actin was shown to interact with fibrin (but not fibrinogen) in a 2:1 stochiometric manner [120]. In vitro experiments provided further support, confirming that the presence of actin in plasma modifies the structure and degradability of fibrin clots, resulting in thinner fibers and delayed plasmin-induced fibrinolysis [46]. Moreover, actin serves as a noncompetitive inhibitor of plasmin by binding to the lysine-binding sites in the kringle domains, effectively obstructing plasmin binding to fibrin [121]. Thus, actin contributes to both clot propagation and fibrinolysis shutdown, through its role in plasmin inhibition. Additionally, it is known that histones, which are rich in positively charged lysine and arginine residues, interact with fibrin and increase resistance to fibrinolysis [122]. The presence of histones in clots alongside fibrin and DNA disrupts the lateral organization of protofibrils, resulting in a fibrin network that exhibits increased resistance to fibrinolysis, despite made up of thick fibers [123]. To summarize, fibrin deposition influences the degradation of necrotic cell debris, as it interacts with components such as actin and histones, potentially hindering the process by increasing resistance to fibrinolysis (Fig. 3).

### The reticuloendothelial system

Although multiple molecules and factors promote systemic clearance of cell debris, the role of the cellular reticuloendothelial system in debris clearance cannot be overstated. A century ago, Aschoff introduced the concept of the reticuloendothelial system, outlining a principle of particle uptake and degradation by both tissue-specific macrophages and endothelial cells of the liver, adrenal gland, pituitary gland, spleen and bone marrow sinusoids [124]. The liver is home to the largest reticuloendothelial system in the body, comprising both the Kupffer cells and the fenestrated liver sinusoidal endothelial cells (LSECs). Kupffer cells are the tissue-resident macrophages of the liver, constituting 80% to 90% of all resident tissue macrophages in the entire body, which highlights their importance. Positioned in the sinusoid vasculature, particularly near the portal areas, Kupffer cells are directly exposed to the circulation and form the first line of hepatic defense by utilizing their array of PRRs to scavenge DAMPs released by dying cells [125]. Upon activation, Kupffer cells release pro-inflammatory cytokines (IL-1β and TNF) and chemokines (CXCL1, CXCL2, CXCL8, and CCL2), resulting in the recruitment of neutrophils and inflammatory monocytes to the site of injury [126]. In parallel, with a total surface area of 210 m^2^ in an adult human liver, the LSECs are responsible for filtering vast amounts of particles passing through the bloodstream. They are the only fenestrated endothelium in the body with open passage between the blood and underlying parenchyma, with the numerous 100–150 nm diameter fenestrations of the LSECs clustered together in “sieve plates”. These facilitate the internalization of a myriad of macromolecules [127, 128]. Of note, not all species have their SECs in the liver, as mammals do. Bony fish have theirs situated in the endocardium of the heart and renal sinusoid, while in cartilaginous fish, lamprey, and hagfish, this is in the gills [129].

**Fig. 4:**
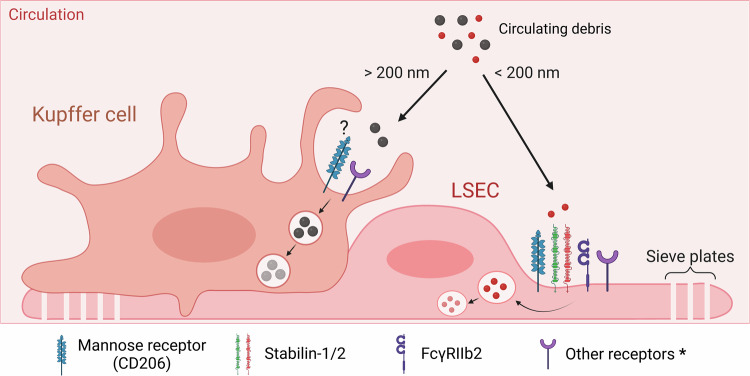
Proposed mechanisms of debris clearance by the hepatic reticuloendothelial system. Circulating debris is internalized through endocytosis (LSECs) or phagocytosis (Kupffer cells) in a size-dependent manner by receptors such as CD206, stabilin-1 and -2 and FcγRIIb2 specifically on LSECs. Subsequently, internalized particles are degraded through lysosomal enzymes. LSEC sieve plates facilitate macromolecule internalization. Conflicting reports exist on CD206 expression on Kupffer cells. *: Other receptors, including those listed in Table 4.

Due to their strategic position within the liver sinusoids and thus close interaction with the circulation, both LSECs and Kupffer cells embody the dual-cell principle of waste clearance, and serve as systemic clearance mechanisms, as illustrated in Fig. 4. More specifically, the LSECs are responsible for clearing bloodborne debris primarily via clathrin-mediated endocytosis (<200 nm), whereas Kupffer cells mostly phagocytose particles larger than 200 nm in size [125, 128]. Moreover, LSECs contain approximately twice the amount of clathrin-coated pits per membrane compared to hepatocytes and Kupffer cells, indicating a high endocytic capacity. This notably high endocytic activity is mediated by scavenger receptors such as stabilin-1, stabilin-2, FcγRIIb2 and the mannose receptor (CD206). Interestingly, both Kupffer cells and LSECs contribute to FcγR-mediated clearance of circulating IgG immune complexes, with LSECs exclusively using FcγRIIb2. Moreover, size dependency has been observed, with a higher number of antibodies complexed to the antigen resulting in an increased direction of the complexes to Kupffer cells [127].

LSECs and Kupffer cells exhibit both complementary and overlapping scavenging activity, and their receptors have already been extensively characterized. However, there are conflicting reports on the expression of CD206 on Kupffer cells, and Kupffer cell homogeneity in general [124, 130]. In healthy murine livers, the existence of two subsets of Kupffer cell populations has been proposed, based on the expression of CD206 and endothelial cell-selective adhesion molecule (ESAM): a major CD206^lo^ESAM^–^ population (KC1) and a minor CD206^hi^ESAM^+^ population (KC2) [131, 132]. However, all genes specifically found in KC2s (*e.g*. CD36) are also expressed by LSECs, suggesting the possibility of cross-contamination between these populations [130, 131]. In contrast, a transcriptome and proteome profiling study in rats found that LSECs and Kupffer cells equally express CD206, but revealed that CD36 was expressed on Kupffer cells while absent on LSECs [133]. Further research is required to elucidate the receptor profiles of Kupffer cells and LSECs. While both cell types have had their receptors and scavenging activity investigated individually, comparative studies considering both components of the dual-cell system remain a rarity. Furthermore, in the context of injury and necrotic cell debris clearance, there is limited knowledge regarding which DAMPs are scavenged by the expressed receptors. Table 4 presents a comparative summary of the receptors expressed by LSECs and Kupffer cells with their respective necrotic cell debris ligands. It is important to note that while the expression of receptors and the binding of ligands have been confirmed, the clearance of necrotic debris ligands by these cells and receptors remains unverified. Once the debris is taken up, the LSECs and Kupffer cells utilize an array of lysosomal enzymes to degrade the endocytosed contents, including but not limited to acid phosphatase, acid lipase, acid DNase (*i.e*. DNase 2), cathepsin D, β-galactosidase, β-glucuronidase and β-acetylglucosaminidase. Interestingly, the high protein content of parenchymal cells gives them higher individual cell enzyme activity than either LSECs or Kupffer cells, though *specific* activities are higher in the latter two. Even between the two, there are stark differences; acid phosphatase, DNase 2 and β-acetylglucosaminidase have increased activity in LSECs, while acid lipase, β-glucuronidase and cathepsin D are more active in the Kupffer cells [134]. The clearance activity of both cell types is highlighted by the fact that lysosomes constitute 6.9% of the volume in LSECs and 13.6% in Kupffer cells. Furthermore, 26% of the total lysosomal volume of an adult rat liver is found in the Kupffer cells, while 17% is found in LSECs [127].

**Table 4:** Receptors expressed on LSECs and Kupffer cells (KCs) with their respective necrotic cell debris ligands.

Receptor	Ligands	Expression on		References
		LSECs	KCs	
FPR1	*N*-formylated peptides	H, M +	H +	[164, 165]
CLEC2D	Nucleosomes	?	H +	[29]
CLEC9A	F-actin	R −	R −	[133]
TLR2	Histones	M +	H, M +	[166, 167]
	HMGB1			
	HSPs			
TLR3	RNA	M +	H, M +	[166, 167]
TLR4	Histones	H, M +	H, M, R +	[140,166–169]
	HMGB1			
	HSPs			
	S100 proteins			
TLR7	RNA	M +	M +	[166]
TLR9	DNA	M +	M +	[166, 170]
SR-A1	HSPs	M, R +	M, R +	[133, 171]
SR-B1	S100 proteins	M, R +	H, R +	[133,172–174]
CD36	Anionic phospholipids	H +, R +/− #	M, R +	[131, 133, 172, 175, 176]
	HSPs			
	S100 proteins			
RAGE	HMGB1	M, R −	H +/−	[174, 177]
	S100 proteins		M, R −	
LRP-1	HSPs	R +	R +	[178, 179]
LOX-1	HSPs	H +	R +/−	[175, 180, 181]
		R −		
SREC-I	HSPs	M +	M +	[171]

+: high expression; −: low expression; ?: not determined; H: human; M: mouse; R: rat. #: + in Wistar rat strain, − in Sprague Dawley rat strain.

Both LSECs and Kupffer cells, essential for maintaining homeostasis, also impact responses to injury. For instance, Kupffer cells have been found to effectively clear circulating chromatin in the liver [135]. Reduction of Kupffer cells is a common feature of liver diseases, such as drug-induced liver injury and ischemia-reperfusion injury [130]. After the acute phase of drug-induced liver injury, Kupffer cells are known to recover through self-renewal and keep doing so during the resolution phase [136]. Generally, Kupffer cells play an important role in the healing and repair process. They promote hepatocyte regeneration and survival through the release of IL-10 and regulate extracellular matrix remodeling through MMP12, MMP13, TIMP2, TIMP3 and ADAM23. Kupffer cells also aid in promoting healing via TIM4-mediated efferocytosis [137]. Studies on mice depleted of Kupffer cells showed hepatic dysfunction and reduced levels of IL-6, IL-10, IL-18-binding protein and complement factor C1q, highlighting their protective role [138, 139]. The loss of Kupffer cells also resulted in disturbances in LSEC homeostasis and integrity, as indicated by increased vascular permeability and hepatic red blood cell accumulation [139]. However, the TLR4 activity of Kupffer cells is a key contributor to the systemic inflammatory response syndrome following drug-induced liver injury. Following acetaminophen overdose, depletion of Kupffer cells, mutation or antagonism of TLR4 reduced mortality and liver injury, and also attenuated expression of pro-inflammatory cytokines (*i.e. Il1b*, *Il6* and *Tnf*). This indicates that the systemic effects due to Kupffer cell activation can exacerbate injury [140]. Furthermore, acetaminophen overdose directly damages the LSECs, causing swelling and destruction of fenestrated pores, which impairs their crucial clearance function and disrupts the sinusoid space [141]. In ischemia-reperfusion injury, research on rats has shown that half of all LSECs lose their functionality after 48 hours of injury, accompanied by a loss of LSEC morphology and alterations in microvascular circulation in the liver sinusoid. The loss of LSEC-mediated local tolerance in homeostasis is also mediated by exposure to the inflammatory environment resulting from Kupffer cell activation and neutrophil infiltration. Finally, upon reintroduction of oxygen to the liver during the reperfusion phase, LSECs greatly increase their scavenging function to cope with the newly produced ROS released by neutrophils [142, 143]. To conclude, injury frequently leads to disruption of the reticuloendothelial system.

## Concluding remarks

The removal of necrotic cell debris generated upon injury is essential for maintaining homeostasis and facilitating tissue healing, as the accumulation of DAMPs can trigger excessive inflammation and potentially lead to autoimmunity. Besides phagocytosis by leukocytes, the body employs several systemic clearance mechanisms, including extracellular DNases, the actin-scavenger system, the fibrinolytic system, and the hepatic reticuloendothelial system. In general, activity of both DNases and the actin-scavenger system is reduced upon injury. The proven efficacy of DNase 1 treatments in murine models warrants further investigation, in conjunction with other mechanism of clearance, such as possible synergism with components of the actin-scavenger system. Furthermore, the therapeutic potential should also be explored in clinical contexts beyond the scope of CF. Given that fibrin can interact with the necrotic cell debris, its role in recovery from injury should be studied in further detail. The same applies to the contribution of plasmin to the innate immune response following injury. Lastly, the scavenging activity of the reticuloendothelial system plays an important role in the clearance of necrotic cell debris. However, more research is needed on their comparative roles rather than their individual contributions and also on the receptors involved.
